# Blue Laser (450 nm) in the Treatment of Port Wine Stains and Telangiectasia

**DOI:** 10.3390/jcm10061258

**Published:** 2021-03-18

**Authors:** Jacek Szymańczyk, Witold Trzeciakowski, Yurij Ivonyak, Piotr Tuchowski, Janusz Szymańczyk

**Affiliations:** 1 Department of Dermatology, Medical University of Warsaw, Koszykowa 82A, 02-008 Warsaw, Poland; szymanczyk.jacek@gmail.com; 2 Institute of High Pressure Physics, Polish Academy of Sciences, Sokołowska 29/37, 01-142 Warsaw, Poland; yi@unipress.waw.pl; 3 Accuro Ltd., Zawodzie 16, 02-981 Warsaw, Poland; p.tuchowski@accuro.pl; 4 Department of Dermatology and Venerology, Medical University of Łódź, 90-643 Łódź, Poland; januszsz7@gmail.com

**Keywords:** port wine stains, blue laser, vascular malformations, laser therapy, facial and leg telangiectasia

## Abstract

This study aimed to test a blue light source for the treatment of port wine stains (PWS) and telangiectasia and to compare this with the application of green and yellow lasers based on data in the literature. A total of 22 patients with PWS were treated with radiation from a novel, high-power 450 nm blue laser that was created for this project. The group contained 15 patients with red PWS and 7 with pink PWS. The best results were achieved for red PWS, using 15–20 ms light pulses and 47 W power. For patients with pink malformations, the results were unsatisfactory. The group with telangiectasia consisted of six patients with facial lesions and three with leg lesions. The recovery was completed for all patients with facial telangiectasia, while the blue laser therapy was ineffective for patients with leg telangiectasia. This study shows that, in some cases, the use of a blue laser may be an alternative to the use of green and yellow lasers.

## 1. Introduction

Port wine stains (PWS) are inborn malformations in the form of pink or red marks, usually located on the face. These lesions are found in 0.3% to 0.5% of newborn children [1]. Attempts to treat PWS and enlarged blood vessels on the face and legs [2] with different lasers started in the early 1980s [3]. However, to date, there is no single laser source that has been 100% effective in the treatment of all cases of PWS. The choice of wavelength for therapy has been limited by the availability of commercial lasers; initially, argon lasers were used, being later replaced by KTP (potassium-titanyl-phosphate) green lasers. At present, the most widely applied and most effective is the pulsed dye laser (PDL), emitting yellow light in the 585–595 nm range. These wavelengths yield fairly large penetration depths [4], while being well absorbed by oxyhemoglobin and deoxyhemoglobin [5,6]; however, the highest absorption for hemoglobin occurs in the blue range (420–450 nm). The absorption by melanin increases in the blue range as well, but not as fast as the absorption of hemoglobin. This range is covered by InGaN/GaN diode lasers, which were developed in the last two decades. Obviously, a high absorption of light implies a rather shallow penetration depth in the tissue. Therefore, it is generally believed that longer wavelengths (i.e., green and yellow) are more effective than shorter blue wavelengths. For the present study, we decided to test this belief. Therefore, we developed a high-power source based on nitride laser diodes and performed clinical tests on patients with various types of PWS and telangiectasia.

## 2. Materials and Methods

### 2.1. Laser Source

**Figure 1:**
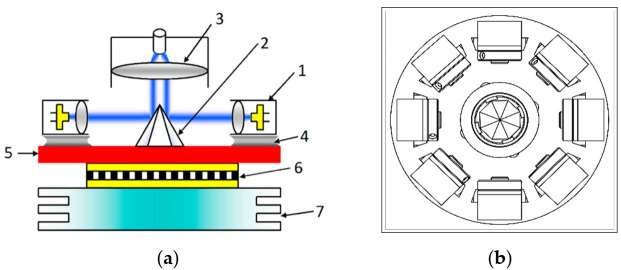
(**a**) Schematic drawing of our fiber-coupling method: laser modules (1) emit blue light (collimated by small lenses, grey), which is then reflected from triangular dielectric mirrors forming an octahedral pyramid (2) and focused into the fiber by the large lens (3). The laser modules are soldered (4) to the copper plate (5), cooled by the Peltier element (6), and placed on a heatsink (7) (reproduced from *Review of Scientific Instruments* 2014, 85, 036106, with the permission of AIP Publishing); (**b**) view from above.

Our laser was designed to fulfil the following requirements: (i) high emission power (5–50 W) for both CW (continuous wave) and pulsed operations, (ii) fiber output, (iii) handpiece allowing for varied spot size, and (iv) built-in power meter. We used laser diodes based on InGaN/GaN heterostructures, which were initially developed for Blu-ray optical discs (at 405–410 nm) and then for laser projectors (at 440–450 nm and 520 nm). Individual diodes in 9 mm packages are now available at 450 nm with power up to 6–7 W. In order to obtain even higher power in an optical fiber, we used the method [7] shown schematically in Figure 1. This method allows for coupling around 90% of the laser light from eight different diodes into the fiber core (0.2 to 0.4 mm in diameter), while ensuring efficient cooling of the diode modules.

**Figure 2:**
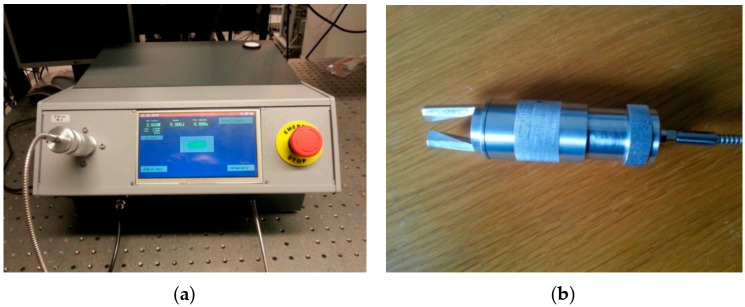
(**a**) The complete blue laser source with a touchscreen, which allows for setting the treatment parameters. A foot pedal is connected at the bottom (black cable). (**b**) The handpiece (applicator) at the end of the fiber. This applicator is inserted into the power meter in the photo on the left.

Each laser diode had an independent power supply, which allows for continuous and pulsed operation (from 1 ms upwards). The plate with eight laser modules (shown in Figure 1) was packed into casing with a touchscreen, as shown in Figure 2. The device was equipped with a laser power meter, which allows for the power to be checked just before treatment. The touchscreen allows for the selection of the following treatment parameters: pulsed or CW mode, beam power (up to 48 W), pulse duration (from 1 ms upwards), time between pulses, number of pulses, and the energy dose per pulse (in J) or the pulse energy density (in J/cm^2^). The handpiece (shown in Figure 2) allows for the size of the light spot on the patient’s skin (from 0.5 to 5 mm) to be adjusted by turning a knob. The device also had some safety features, such as a door blockade and emergency switch.

We also developed a similar laser at 520 nm (green emission), but with lower power (5 W). The setup shown in Figure 1 allows for the mixing of up to eight different wavelengths in the fiber and for simultaneous illumination with a few colors [8]. In the present study, we focused on clinical tests using the pure blue source.

### 2.2. Patients and Treatment Method

The project received the approval of the Bioethical Commission of the Medical University of Warsaw (Approval Code KB/212/2015). A total of 22 patients with port wine stains (PWS) and 9 patients with telangiectasia were treated using the 450 nm laser source described above. The treatments started in May 2016 and were completed in September 2020. All clinical work was performed at the Dermatology Clinic of Warsaw Medical University. The patients signed the treatment agreement form and were checked for the following contraindications: pregnant or breastfeeding women, patients with phototoxic or photoallergic reaction, epilepsy, cancer and precancerous lesions, patients with a strong sunburn, and patients with herpes simplex in the treated area.

There were 7 patients with pink PWS, 15 with red PWS, 6 patients with facial telangiectasia, and 3 with leg telangiectasia. In Table 1 and Table 2, we list the data for all the patients with red and pink PWS, respectively. Irradiations were performed at one-month intervals, or once every two months (in periods of increased sun exposure). In total, there were 231 treatments (98 for patients with pink PWS and 133 for patients with dark red PWS). Therefore, on average, each patient with PWS received 10.5 treatments. In the group of patients with telangiectasia, we performed 34 treatments.

The irradiations were performed using the handpiece shown in Figure 2, adjusting the light-spot size at the end of the fork. In most cases, the selected spot diameter was 2 mm, since the power density seemed too low at 5 mm diameter. This means that the treatments were long (a scanner would be useful). Around 2 cm^2^ of the PWS was irradiated in each treatment. Initially, we used lower powers and longer pulse durations, such as 9 W for 200 ms or 20 W for 50 ms. However, as shorter times led to better results, we switched to 47 W with a 15–20 ms pulse duration. These parameters seemed to be optimal. In the group of patients with telangiectasia, we started with 9 W laser power and up to 160 ms pulses. However, as higher powers (and shorter pulse lengths) turned out to be more effective in the PWS treatments, we decided to apply 47 W power with 10–15 ms pulses.

The irradiations turned out to be rather painful for the patients, so we used local anesthetic in the form of 5% EMLA (lidocaine and prilocaine) cream and cooled the tissue with a flow of air. The temperature of air in the cooler was −30 °C, but the typical temperature of cooled skin was 6–8 °C. The local cooling reduced the pain, and the evacuation of heat reduced the risk of scarring.

## 3. Results

**Figure 3:**
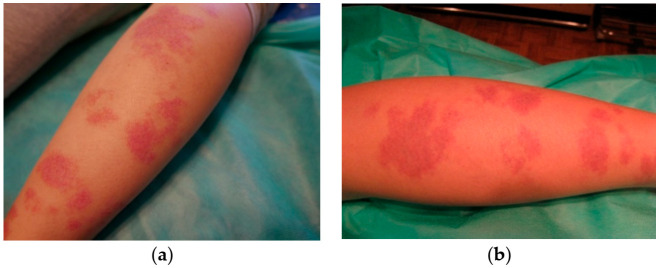
Pink PWS on legs with very poor results (estimated at 2 points) following therapy: (**a**) before treatment and (**b**) after treatment.

**Figure 4:**
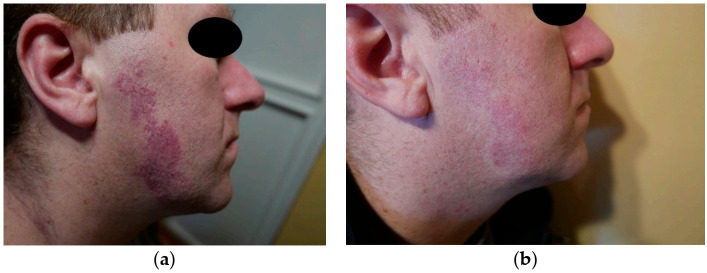
Red PWS on the cheek: (**a**) before treatment and (**b**) after treatment (estimated at 9 points).

**Figure 5:**
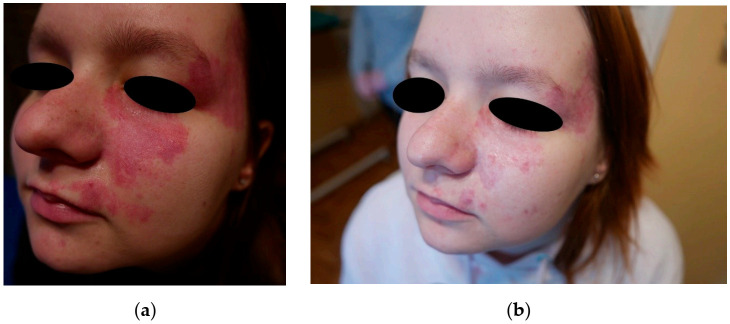
Red PWS on the face: (**a**) before treatment and (**b**) after treatment (estimated at 5 points).

**Figure 6:**
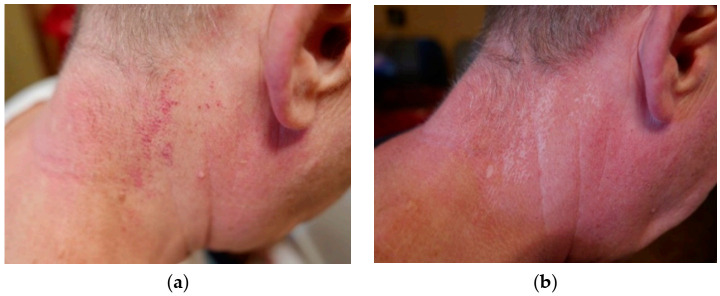
Red PWS on the neck: (**a**) before treatment and (**b**) after treatment (estimated at 9 points).

In order to quantify the effects of the treatment, we introduced a scale from 1 to 10 describing the results subjectively. This was mainly based on the color of the lesions after treatment. For the group of seven patients with pink PWS, only one patient had a lesion of lighter color (7 points), two were estimated at 4 points, one at 3 points, and one at 2 points. Two patients gave up treatment before it was completed. In Figure 3, we show the results for pink PWS that we considered ineffective based on the lack of discernible changes. For the group of 15 patients with red PWS, 6 patients had significantly eliminated lesions (9 points), 2 were given 7 points, 3 were given 5 points, and 1 was estimated at 3 points. Three patients with red PWS gave up treatment before completion. Some examples are shown below (Figure 4, Figure 5 and Figure 6).

The therapy was fairly effective on the cheek, as can be seen through reduction in the intensity of red PWS in Figure 4 and Figure 5. The results were slightly worse for red PWS on the temple (Figure 5).

In Figure 6, we can see quite good overall results for the removal of red PWS on the neck, although some depigmentation can be observed.

We summarize our results in Table 3. We calculated the success rate of treatment as the sum of points achieved for each group divided by the maximum number of points. Taking into account only the fully treated patients (5 for pink PWS and 12 for red PWS), we obtained 7 + 8 + 5 = 20 points for pink PWS and 54 + 14 + 15 + 3 = 86 points for red PWS. This yielded a 20/50 = 40% success rate for pink PWS and a 86/120 = 71.7% success rate for red PWS.

**Figure 7:**
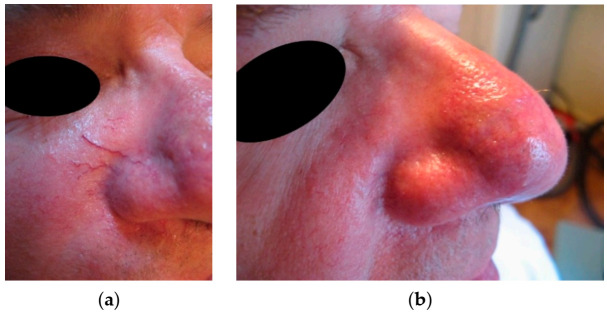
Telangiectasia on the nose: (**a**) before treatment and (**b**) after treatment.

In the case of the six patients with facial telangiectasia, the recovery was complete in all cases (see the example in Figure 7), while for the three cases of leg telangiectasia, the therapy was ineffective.

## 4. Discussion

Laser therapy is currently the standard method for treating PWS. However, such treatment leads to complete elimination of lesions in only 15–20% of cases [9]. There seem to be three main factors affecting the results of laser therapy: The first is the depth of the enlarged blood vessels. Many studies have shown a correlation between the depth of lesions and the response to laser therapy [10]. When the depth of the lesion exceeds 1030 μm, the results of treatment are poor. In the case of more superficial lesions (below 830 μm), the response to treatment is generally much better [11]. The second factor influencing the results seems to be the size of the enlarged blood vessels. Pathologically enlarged vessels can be divided into four groups: The first group corresponds to vessels below 80 μm in diameter, observed clinically as light pink marks. The second group is represented by vessels up to 120 μm in diameter, observed as darker pink marks. The third group includes vessels up to 150 μm in diameter, leading to red marks. Vessels above 150 μm belong to the fourth group and give rise to purplish marks and papules [12,13,14]. According to many authors, an increase in laser pulse duration can lead to better results for larger vessels. This follows from the calculated dependence of the thermal relaxation time on the size of the blood vessel [15].

The third factor affecting the results of laser therapy is cooling of the treated area. There are several methods of cooling. Most authors have raised the following advantages brought by cooling, either before or during laser illumination [16]: (i) reduction of the thermal damage to the epidermis, (ii) reduction of pain, and (iii) lower risk of scarring [17].

The most common laser source used for PWS treatment is a pulsed dye laser (PDL), and most published results are based on the application of PDLs. The first generation of these lasers (produced in 1980) produced emissions of 577 nm wavelength, while the new generation emits in the 595–600 nm range. The pulse duration of this device is in the 0.45–40 ms range.

PDL treatment is not effective for small and deep PWS [18]. In general, around 20–30% cases resist treatment. A retrospective study of a group of 261 patients with flat PWS treated with 595 nm PDL [19] has shown that lesions located on the cheek, neck, and forehead react to therapy much better than those located around the mouth, eyelid, or nose. For patients with dark red lesions (purple group), the clearance rate was significant (53.5%), while for patients with pink lesions (red group), it was much lower (36.1%). The study showed that illumination with short pulses (0.45, 1.5, or 3 ms) led to a better clearance rate than applications using longer pulses (i.e., 6 ms or more). There is also some evidence [20] that using multiple pulses of PDL with lower fluence may be more effective than using a single pulse with higher fluence.

Another laser frequently used in PWS therapy is the frequency-doubled KTP laser, emitting 532 nm of green light. Similarly to yellow light at 595 nm, this wavelength is well absorbed by oxyhemoglobin but, unfortunately, even more strongly by melanin. This affects the penetration depth [21] and increases the probability of scarring. Due to the smaller penetration depth, these lasers are most efficient for treating superficial vascular lesions, including capillary malformations. KTP lasers have shown improved treatment results for previously untreated and treatment-resistant lesions [22].

In the case of enlarged blood vessels on the face (facial telangiectasia), both KTP (532 nm) and PDL (585 nm) have been used [2]. Clinical results for these two lasers have turned out to be similar, but the pain level was higher with the yellow laser (PDL) compared to the green one (KTP). Equally positive results have been obtained in the treatment of telangiectasia with PDL (595 nm) and with a filtered IPL (Intense Pulsed Light) lamp for the 530–650 nm range and 900–1200 nm (infrared) range [23]. The only side effect when applying the above light sources was local swelling and redness, although without any scarring.

It is finally worth mentioning the oldest laser source used for the treatment of PWS and telangiectasia (since 1970); namely, the argon ion laser emitting in the blue–green range (two strongest lines at 488 and 514 nm). These wavelengths are strongly absorbed by oxyhemoglobin [24]. However, the CW (or quasi-CW) operations of the Ar laser resulted in a high probability of scarring and high risk of hyper- or hypopigmentation. The appearance of new laser sources emitting different wavelengths and allowing for pulsed operations has resulted in the Ar laser being eliminated from common use.

Taking into account the available data in the literature, we found that our 450 nm laser source can provide an effective alternative for the laser therapy of PWS (especially for red PWS) and facial telangiectasia.

## 5. Conclusions

1. Our clinical studies showed that for the 450 nm blue laser, the best therapeutic and cosmetic results were achieved with 47 W power, 15–20 ms pulses, and 2 mm spot diameter. The observed results seem comparable to those obtained for PDL lasers with shorter pulse durations.
2. Better results in terms of more effective lightening of marks were obtained for red PWS compared to pink PWS. This is probably due to the deeper location of enlarged blood vessels for pink PWS and, therefore, the lower light intensity at these depths.
3. The blue laser turned out to be effective for the treatment of facial telangiectasia.
4. High emission power and short pulses seem to reduce thermal injury and the risk of scarring. Therefore, research into the further reduction of pulse duration (with a simultaneous increase in power) seems promising for future studies. Another interesting possibility is the use of multiple pulses with lower fluence [20]. This might allow for an increase in the laser spot size during treatment.

## Figures and Tables

**Table 1:** Data for the patients with red port wine stains (PWS).

Patient’s Number	Sex	Age	Type of Skin	Localization	Size (cm^2^)	Number of Procedures	Evaluation of Effects
1	F	74	II	Back	4.5	10	9/10
2	M	40	II	Right ear	8	17	5/10
3	M	57	III	Neck and left temple	65	26	7/10
4	F	45	II	Thigh right	5	10	9/10
5	M	26	III	Forehead	7	12	9/10
6	F	63	III	Chin and right cheek	150	18	3/10
7	M	58	II	Neck	5	2	9/10
8	F	36	III	Left temple	6	4	7/10
9	M	53	III	Left part of forehead	5	1	Resignation after one procedure
10	F	49	IV	Left mandibular arch	2	2	Resignation after two procedures
11	F	64	II	Left arm	2	3	9/10
12	F	62	III	Left calf	12	3	5/10
13	M	39	III	Right cheek	25	10	9/10
14	F	34	II	Chest	72	2	Resigned due to pregnancy
15	F	15	III	Left cheek and temple	30	13	5/10

**Table 2:** Data for the patients with pink PWS.

Patient’s Number	Sex	Age	Type of Skin	Localization	Size (cm^2^)	Number of Procedures	Evaluation of Effects
1	F	38	II	Left cheek	60	11	4/10
2	F	51	II	Left cheek and left part of forehead	18	22	4/10
3	F	24	II	Right leg	10	10	Resigned due to pregnancy
4	M	54	III	Behind right ear, right cheek and neck	100	32	3/10
5	M	30	III	Right part of face	50	14	2/10
6	F	32	III	Left shank	40	6	7/10
7	F	44	II	Left foot	4	3	Resigned after three procedures

**Table 3:** Estimated results of treatment.

	Number of Patients	Number of Fully Treated Patients	Sum of Points	Success Rate
Pink PWS	7	5	20	40%
Red PWS	15	12	86	71.7%
Total	22	17	106	62.4%

## Data Availability

No publicly archived datasets.
